# Chloramphenicol acetyltransferase as a selection marker for chlamydial transformation

**DOI:** 10.1186/1756-0500-6-377

**Published:** 2013-09-23

**Authors:** Shuang Xu, Lauren Battaglia, Xiaofeng Bao, Huizhou Fan

**Affiliations:** 1 Department of Pharmacology, Rutgers University Robert Wood Johnson Medical School, Piscataway, NJ, USA; 2 Rutgers University Graduate Program in Cell and Developmental Biology, Piscataway, NJ, USA; 3 Department of Pharmacology, Nantong University School of Medicine, Nantong, China

**Keywords:** *Chlamydia*, *Chlamydia trachomatis*, Chloramphenicol, Chloramphenicol acetyltransferase, Sexually transmitted infections, Transformation, Transformation selection marker

## Abstract

**Background:**

*Chlamydia* is a common bacterial pathogen responsible for many diseases. Methods for transforming this important organism using a β-lactamase as a selection marker have been developed very recently. However, the National Institutes of Health Guidelines for Research Involving Recombinant DNA Molecules do not permit transformation experiments with β-lactamase gene-containing vectors for certain human chlamydial pathogens. Therefore, a different selection marker is urgently needed for transformation of those chlamydiae.

**Results:**

After transformation of plasmid-free *Chlamydia trachomatis* with pGFP:SW2, which carries a β-lactamase and a chloramphenicol acetyltransferase gene fused to a green fluorescence protein gene, transformants were obtained by selection with either ampicillin or chloramphenicol. Stable chloramphenicol-resistant, but ampicillin-sensitive, transformants were obtained using a pGFP:SW2 derivative without the β-lactamase. All transformants expressed green fluorescence protein and had glycogen synthesis activity restored.

**Conclusions:**

Chloramphenicol resistance may be used as a selection marker for genetic experiments in *Chlamydia*. This eliminates the requirement for the use of β-lactamase, of which dissemination to some *C*. *trachomatis* serovars may jeopardize clinical treatment of chlamydial infections in pregnant women. Chloramphenicol acetyltransferase may also serve as a useful secondary selection marker for genetic analyses in β-lactamase-transformed chlamydial strains.

## Background

*Chlamydia* is a bacterium that replicates only inside a eukaryotic host cell, and is a causative agent for a number of human diseases [1,2]. Acquired by sexual transmission, replication of *Chlamydia trachomatis* at the lower genital tract causes cervicitis [1]. Subsequently, chlamydiae ascend to the upper genital tract, causing inflammation leading to tissue disruption and scarring in the uterus and oviducts. Inflammation in the uterus during pregnancy may lead to abortion and premature birth, whereas chlamydial oviductal pathology constitutes the number one cause of female infertility, and ectopic pregnancy [1]. Furthermore, an infant of an infected mother may acquire the pathogen while passing the birth canal, and develop respiratory infection [1]. *C*. *pneumonia* is a common pathogen of community-acquired respiratory infection, and a co-factor for arteriosclerosis and late-onset Alzheimer disease [3-5]. At the present time, no vaccine effective against human chlamydial diseases or other preventive means are available [6,7].

*Chlamydia* has a unique developmental cycle, which consists of two alternating cellular forms: the infectious but non-dividing elementary body (EB) and the dividing but non-infectious reticulate body (RB). Developmental events including conversion of EB to RB, replication of RBs, and RB to EB reorganization take place inside a cytoplasm vacuole designated inclusion. For a long period of time, study of *Chlamydia* has been severely hampered by a lack of a convenient genetic system. Fortunately, a method for transforming *C*. *trachomatis* with shuttle vectors using CaCl_2_ has been developed very recently by Wang et al. [8], and has been adopted by others [9,10]. Furthermore, Gérard et al. have transformed *C*. *pneumoniae* using dendrimers as a delivery tool [11]. Both these methods were established with plasmids carrying a β-lactamase, which degrades penicillin family antibiotics, and thus relieves their inhibitory effects of these drugs on the cytokinesis of RBs [8,9]. Since amoxicillin, a penicillin family member, is recommended for treatment of chlamydial cervicitis in pregnant women, transformation experiments using β-lactamase marker for sexually transmitted, non-LGV chlamydial pathogens (i.e., *C*. *trachomatis* serovar D to K), are not permitted by the National Institutes of Health Guidelines for Research Involving Recombinant DNA Molecules. Therefore, there is an urgent need to identify selection markers for recombination studies in those chlamydial serovars. In addition, there is also a need to identify secondary selection markers for chlamydiae which may already carry an established resistant marker such as a β-lactamase for different reasons in future studies. Here, we report the identification of a chloramphenicol acetyltransferase (CAT) gene as a useful selection marker.

## Methods

### Tissue culture and molecular biology reagents

Dulbecco’s modified Eagle’s medium (DMEM) with high glucose (4.5 g/L) and 110 mg/L sodium pyruvate, fetal bovine serum (FBS), Dulbecco’s phosphate-buffered saline (PBS), trypsin-EDTA solution for cell culture, Trizma base, cycloheximide, CaCl_2_, ampicillin, chloramphenicol and Lugol solution were purchased from Sigma Aldrich. Gentamycin and transformation-competent *Escherichia coli* stbl2 cells were purchased from Invitrogen. *Pfu* Ultra DNA polymerase was purchased from Agilent. *Bam*H1 and the Q5 Site-Directed Mutagenesis Kit were purchased from New England Biolabs.

### Vectors

**Figure 1:**
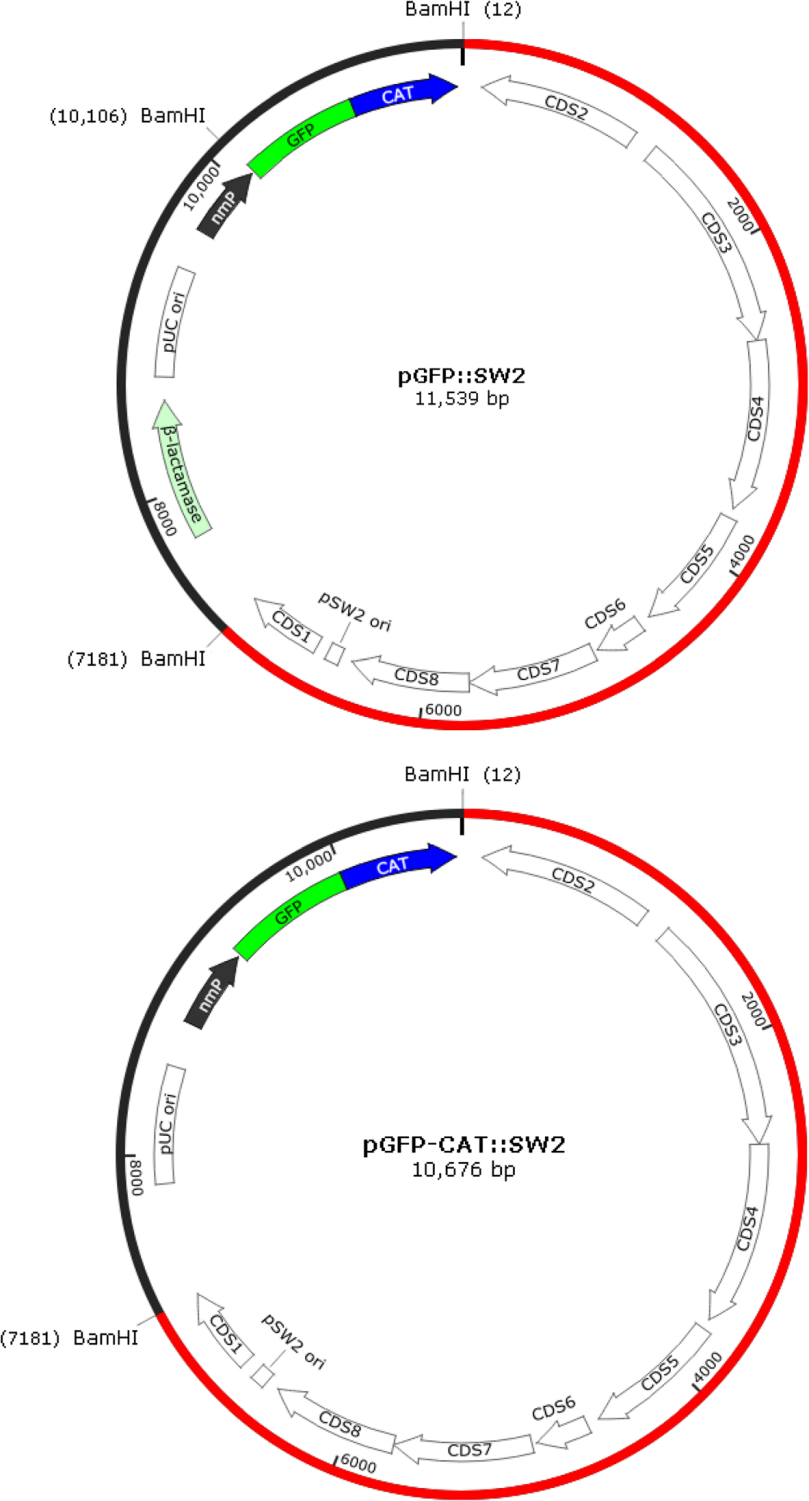
**Schematic presentation of pGFP::SW2 (top) [**[8]**] and its derivative pGFP-CAT::SW2 (bottom).** Sequence originated from the *C*. *trachomatis* SW2 plasmid is shown in red, and non-chlamydial sequences in black. Compared to pGFP::SW2, the β-lactamase gene has been removed and a *Bam*H1 cutting site located upstream of the GFP-CAT fusion gene has been inactivated in pGFP-CAT::SW2. All other features, including the *C*. *trachomatis* plasmid replication origin (pSW2 ori) and coding sequences (CDS) 1-8, *E*. *coli* pUC replication origin (pUC ori), the GFP-CAT fusion gene and the *Neisseria meningitidis* promoter (nmP) that drives the expression of GFP-CAT, remain unchanged in pGFP-CAT::SW2. See Methods for construction details.

pGFP::SW2 is a shuttle vector containing an *E*. *coli* replication origin, a β-lactamase gene, a chloramphenicol acetyltransferase gene fused to DNA coding for a red-shifted green fluorescence protein (GFP), and nearly the complete sequence of a *C*. *trachomatis* plasmid designated SW2 (Figure 1, top) [8]. pGFP-CAT::SW2 (Figure 1, bottom) was derived by deletion of the β-lactamase gene from pGFP::SW2. Briefly, the 7169 bp SW2 DNA was removed from the plasmid by first amplifying the non-chlamydial DNA sequence using the *Pfu* Ultra DNA polymerase and a pair of mutually complementary primers, GFP-5’c (5’TAGATTAATGTCGACTCTAGAGGATCCGGGTACCGAGCTCGAA3’) and GFP-3’c (5’TTCGAGCTCGGTACCCGGATCCTCTAGAGTCGACATTAATCTA3’), which target the termini of the non-chlamydial DNA fragments, and then circularization of the fragment in *E*. *coli*. Likewise, the *Bam*H1 site within the GFP coding sequence of the resulting 4,370 bp plasmid, designated pBETA-GFP-CAT, was inactivated using the mutually complementary primers GFP-Del-BamH1-5’c (5’AAGCCGATATGGTGGATTCCCGGGTACCAATGA3’) and GFP-Del-BamH1-3’c (5’TCATTGGTACCCGGGAATCCACCATATCGGCTT3’). The 861 bp β-lactamase open reading frame was removed from the plasmid using the Q5 Site Directed Mutagenesis Kit to yield the plasmid pGFP-CAT. Primers for Q5-mediated mutagenesis were Del-amp1 (5’CTGTCAGACCAAGTTTAC3’) and Del-amp2 (5’ACTCTTCCTTTTTCAATATTATTG3’). Finally, the 7168 bp SW2 fragment was inserted to the *Bam*H1 site of the pGFP-CAT to yield pGFP-CAT:SW2 (Figure 1). Chloramphenicol was used to select *E*. *coli* transformed with pGFP-CAT and pGFP-CAT:SW2 (Figure 1). Sequence authenticity of the SW2 portion of pGFP-CAT::SW2 was confirmed through paid service provided by Macrogen USA.

### Plasmid DNA preparation

*E*. *coli* stbl2 cells were used to grow plasmids. Plasmid DNA was purified using Qiagen Midi DNA purification columns, precipitated by ethanol and redissolved in 10 mM Tris-HCl (pH 8.5). DNA concentration of final preparations was 2.5 μg/μl or above.

### Cells and culture conditions

HeLa and McCoy cells were purchased from American Type Culture Collections (ATCC, Manassas, VA), and maintained as adherent cultures in 5% CO_2_ incubators with 100% humidity using DMEM containing 10% FBS [8].

### Preparation of EBs for transformation

EBs for transformation experiments were prepared as previously described [8] with minor modification. Five T150 flasks of 95% confluent HeLa cells were inoculated with an EB stock of the plasmid-free *C*. *trachomatis* 2566R (L2R) [12] at a multiplicity of ~5 inclusion-forming units (IFU) per cell, and cultured with medium containing 20 μg/ml gentamycin and 1 μg/ml cycloheximide. 46 h after inoculation, the infected monolayers in each flask were briefly rinsed with 5 ml PBS, and 3 ml of trypsin-EDTA. When cells appeared to have rounded up under an inverted microscope, they were collected into 8 ml DMEM-FBS. Cells from all 5 flasks were pooled, and subjected to centrifugation at 500 *g* for 10 min. Following the removal of the supernatant, cells were resuspended in 1 ml SPG buffer [13] and disrupted by three cycles of 5-s on/5-s off sonication using a Sonic Ultrasonic Processor (Model number: GEX 130) equipped with a 3-mm diameter stepped microtip. The energy intensity level of the sonicator was set at 40% [14]. The cell lysate was centrifuged at 750*g* for 10 min. Aliquots of the supernatant, which contained EBs, were made and stored at -80°C. The titer of this batch of EB stock was determined to be 2 × 10^9^ IFU/ml.

### Transformation and selection of transformants

Transformation was carried out as previously described [8] with modifications. 7.5 μl of the above frozen L2R EB stock was combined with 10 μg plasmid DNA in 3.5 μl volume and 189 μl 50 mM CaCl_2_ buffer (prepared with 10 mM Tris, pH 7.4) in a 1.5 ml tube, which was let stand at room temperature for 40-45 min. McCoy cells of a 20 h culture at 80% confluence in a 145-mm diameter plate were trypsinized, collected into 14 ml antibiotic-free DMEM-FBS, and centrifuged at 500 *g* for 5 min. Following the removal of the supernatant, the cell pellet was rinsed gently with 10 ml of PBS, resuspended with 14 ml PBS, and centrifuged again. The pellet was resuspended in 220 μl CaCl_2_ buffer. 200 μl of the cell suspension was then added into the plasmid-EB-CaCl_2_ mix. The final transformation mix was incubated at room temperature for 20 min, during which the tube was shaken from time to time to prevent settling of the cells. At the end of the 20 min incubation period, the cells were diluted with 20 ml DMEM-FBS, and plated onto four T25 flasks (5 ml/flask) and were cultured at 37°C under 5% CO_2_ and 100% humidity.

Selection of transformants was initiated 10 h after the initial plating of the transformation. Ampicillin and chloramphenicol were added to the cultures to yield a final concentration of 2 μg/ml and 0.1 μg/ml, respectively. This initial passage was defined as passage 1. 46 h later, cells in the T25 flasks were harvested and disrupted by sonication. The entirety of the P1 culture was passed onto a new T25 flask of McCoy cells at 80-90% confluence. The P2 flasks were cultured with DMEM-FBS containing 1 μg/ml cycloheximide plus 5 μg/ml ampicillin or 0.2 μg/ml chloramphenicol. Passage and selection were repeated every 48 h for two additional passages through passage 4. 90% harvest of passage 4 was frozen and the remaining was expanded with medium containing 5 μg/ml ampicillin or 0.5 μg/ml chloramphenicol.

### Glycogen stain

To stain glycogen in chlamydial inclusions, the culture medium was replaced with Lugol solution, which contains iodine, at 40 h after inoculation.

### Imaging acquisition and processing

All phase contrast and GFP images were obtained at 40 h post inoculation from live cultures in PBS supplemented with 1.3 mM CaCl_2_, 1.0 mM MgCl_2_ and 1.0 g/L (w/v) glucose using an Olympus IX-51 microscope and an Olympus monochrome CCD camera. Images processing (coloring and color overlay) were accomplished by using the Picture Frame software [15,16].

## Results and discussion

pGFP::SW2, the shuttle vector constructed by Wang et al, contained a β-lactamase gene as a selection marker. It also carries a CAT gene that is fused to the GFP gene [8]. We found that *E*. *coli* transformed with the pGFP::SW2 plasmid was able to grow in medium containing chloramphenicol (final concentration: 170 μg/ml), suggesting that the CAT gene may also be used as a selection marker for chlamydial transformation experiments. As cautioned by Wang et al., an adverse effect of chloramphenicol on host mitochondria, as demonstrated by Li et al. [17] may affect the utility of this antibiotic for chlamydial transformation [8]. In Li et al.’s report, the minimal toxic concentration of chloramphenicol was 10 μg/ml, which caused a mild 20% reduction of ATP production. We have determined that the apparent lowest chloramphenicol concentration that completely inhibited inclusion formation in the plasmid-free *C*. *trachomatis* L2 strain designated L2R was only 0.05 μg/ml (data not shown), which was far below the concentrations toxic to host cells. Therefore, we reasoned that this antibiotic could be used to select CAT-expressing transformants without significantly stressing host cells.

We transformed L2R with pGFP::SW2, and selected one half of the transformed cells with ampicillin, and the other half with chloramphenicol. The antibiotics (2 μg/ml ampicillin or 0.1 μg/ml chloramphenicol) were added to the cultures at 10 h after transformation. Starting at passage 2, their concentrations were increased to 5 and 0.2 μg/ml, respectively, and they were added at the time of inoculation. The splitting ratio between passages remained 1:1 from passage 1 though passage 4. Even though the MIC of chloramphenicol, which was determined at low multiplicity of infection (~0.1 inclusion-forming units per cell), was 0.05 μg/ml, some of the untransformed chlamydiae were expected to form inclusions in the presence of 0.1 - 0.2 μg/ml the antibiotic, because we used a high EB to cell ratio for transformation, and efficacy of chlamydial growth inhibition by antibiotics is influenced by the multiplicity of infection [18]. With the above described selection protocol, in cultures selected with ampicillin, under a phase-contrast microscope, nearly 100% cells were found to contain an inclusion with aberrant RBs at the initial passage. The aberrant RB-containing inclusions were still present in a small proportion of cells at passage 2, but were mostly replaced by apparently normal inclusions at passage 3. Normal inclusions were found in about 20% cells, and abnormal inclusions were rarely observed at passage 4. At passage 5, which resulted from inoculation with 10% harvest from passage 4, inclusions were found in 80% cells; apparently, none of these inclusions contained aberrant RBs.

Since chloramphenicol does not cause chlamydiae to form aberrant RBs, we judged resistance to the antibiotic by the inclusion size. Inclusions of smaller-than-normal sizes were detected in almost all cells at passage 1. Some, but very small-sized, inclusions were found at passage 2, and most of those inclusions were replaced with normal-sized inclusions by passage 3. Normal-sized inclusions were found in more than 20% cells at passage 4. At passage 5, derived after inoculation with 10% passage 4 harvest, and an increase in chloramphenicol concentration (from 0.2 μg/ml to 0.5 μg/ml), normal-sized inclusions were found in nearly 100% cells 0000000.

**Figure 2:**
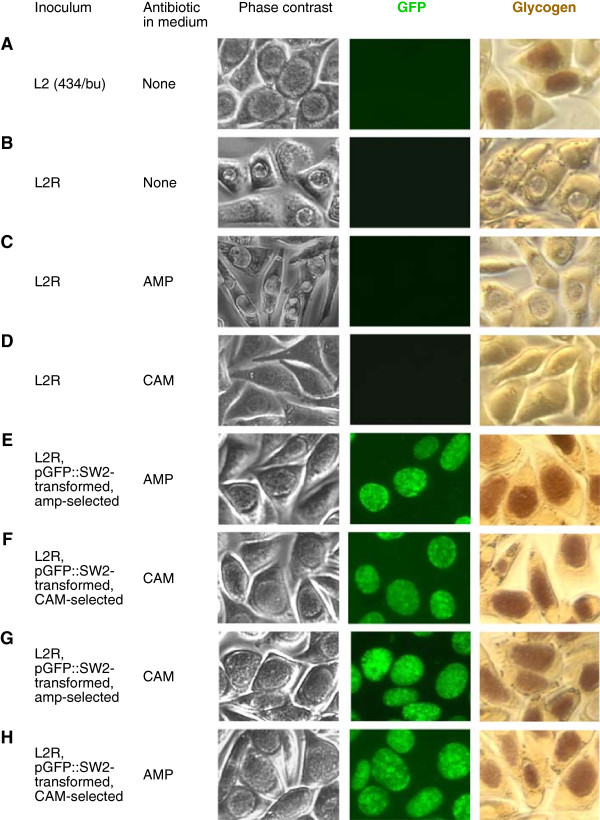
**Inclusion, GFP expression and glycogen synthesis of pGFP::SW2 transformants of *C*. *trachomatis *L2.** Untransformed plasmid-replete strain 434/bu **(A)**, untranformed plasmid-free strain L2R **(B-D)**, and transformed L2R selected with either ampicillin **(E, G)** or chloramphenicol **(F, H)** were cultured either with medium containing an indicated antibiotic or with antibiotic-free medium. Images of inclusions in unstained and iodine-stained cultures were acquired with phase contrast microscopy or fluorescence microscopy 48 h post-inoculation. In all panels, phase contrast and GFP images are superimposable.

In addition to apparent resistance to the selection agents, we used GFP expression and restoration of glycogen synthesis as additional markers for successful transformation [8,9,19]. EBs harvested from passage 5 were used to infect McCoy cells at a 1:100 dilution rate (cell number equivalence) for experiments determining GFP expression and glycogen synthesis (Figure 2). As expected, neither wild-type plasmid-replete *C*. *trachomatis* L2 (434/bu) (Figure 2A) nor untransformed plasmid-free L2R (Figure 2B) expressed GFP. Consistent with established findings that glycogen synthesis in *C*. *trachomatis* requires its plasmid [8,9,19,20], iodine stain showed that glycogen was accumulated in the inclusions of wild-type L2 (Figure 2A) but not those of L2R (Figure 2B). In the presence of 5 μg/ml ampicillin, which inhibits RB division, non-transformed L2R (Figure 2C) formed inclusions containing giant RBs without producing glycogen; whereas chloramphenicol (final concentration: 0.5 μg/ml) completely inhibited the formation of visible inclusions in L2R infected-cells (Figure 2D). Consistent with data published by Wang et al. [8], pGFP::SW2-transformed L2R selected with ampicillin formed GFP-positive inclusions with glycogen (Figure 2E). pGFP::SW2-transformed L2R selected with chloramphenicol also formed GFP-positive inclusions containing glycogen (Figure 2F). As expected, ampicillin-selected transformants were able to form normal-sized, GFP-positive, glycogen-containing inclusions in the presence of chloramphenicol (Figure 2G); likewise, chloramphenicol-selected transformants were completely resistant to ampicillin, expressed GFP and produced glycogen (Figure 2H). These results suggest that the CAT gene in the pGFP::SW2 plasmid is functional in chlamydiae, and chloramphenicol-resistance can be used as a selection marker for chlamydial transformation.

**Figure 3:**
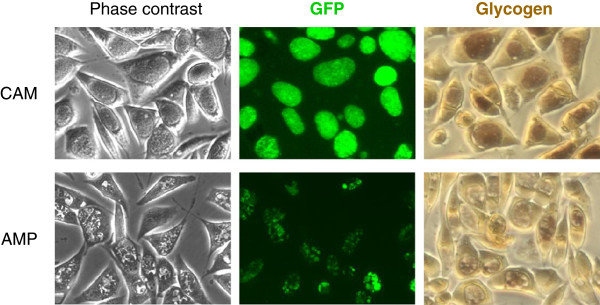
**Inclusion**, **GFP expression and glycogen synthesis in L2R transformed with pGFP**-**CAT::SW2.** Cells infected with chloramphenicol-selected transformants were exposed to either chloramphenicol or ampicillin. Images of inclusions in unstained and iodine-stained cultures were acquired with phase contrast microscopy or fluorescence microscopy 48 h post-inoculation. Phase contrast and GFP images are superimposable.

We next removed the β-lactamase gene from the pGFP::SW2 plasmid as described in the "Methods" section, and used the resulting pGFP-CAT::SW2 plasmid to transform L2R. Transformed chlamydiae were subjected to selection with chloramphenicol using the same regimen as described above. Chloramphenicol-resistant inclusions emerged in about 20% of the infected cells in the culture of passage 4. Fluorescence microscopy and iodine stain revealed GFP- and glycogen-positive inclusions in cells that were infected with EBs harvested from passage 5, and cultured in the presence of chloramphenicol (Figure 3, upper panel). However, as a result of a lack of β-lactamase in the pGFP-CAT::SW2 plasmid, the transformants were not able to form normal inclusions in the presence of ampicillin; instead, their inclusions were filled with aberrant RBs. While the irregular inclusions were still positive for GFP, they were largely free of glycogen (Figure 3, lower panel). After passage 5, the pGFP-CAT::SW2 transformants were cultured with 0.5 μg/ml chloramphenicol for additional four passages at a 1:100 splitting ratio for each passage. All inclusions at passage 9 had the same appearance as the inclusions of plasmid-replete chlamydiae and not that of the plasmid-free L2R, and all were found to express GFP (data not shown). These results prove that the CAT gene in the pGFP-CAT::SW2 plasmid, similar to the pGFP::SW2 plasmid, functions as a selection marker for *C*. *trachomatis* transformation.

It should be noted that Tam et al. has previously attempted to develop a transformation system with the CAT gene as a selective marker [21]. In that study, a shuttle vector containing a CAT gene was electroporated into EBs of *C*. *trachomatis* E (strain UW-5/CX) and L2 (strain 434/Bu). Although both CAT mRNA and enzyme activity were detectable in the initial cultures of transformed chlamydiae, the authors failed to obtain stable transformants after selection with chloramphenicol for 4 passages. It is important to note that both strains electroporated contain a native plasmid that shares the same replication origin with the recombinant plasmid used for transformation [21]. Since Wang et al. were able to obtain stable penicillin-resistant transformants only from L2R, a plasmid-free *C*. *trachomatis* L2 variant, but not the wild-type, plasmid-replete 434/Bu, under otherwise identical experimental settings [8], it is retrospectively not surprising that Tam et al. failed to derive stable chloramphenicol-resistant chlamydiae.

## Conclusions

We have shown that for the plasmid-free *C*. *trachomatis* L2R, chloramphenicol can be used to select transformants when a shuttle vector that carries a CAT gene is used. Since Wang et al in Europe were able to select transformants of a clinical strain of serovar F with penicillin [19], CAT gene should be a highly useful and legally compatible selection marker for serovar F and other *C*. *trachomatis* serovars for which use of β-lactamase-carrying vectors are currently not permitted in the US. Furthermore, we envision that the CAT gene may also serve as a valuable secondary marker for conducting gene-targeting experiments and other genetic experiments such as in *C*. *trachomatis* lymphogranuloma strains that (will) have already been transformed with a β-lactamase-bearing plasmid.

## Abbreviations

AMP: Ampicillin; EB: Elementary body; CAM: Chloramphenicol; CAT: Chloramphenicol acetyltransferase; DMEM: Dulbecco’s modified Eagle’s medium; EDTA: Ethylenediaminetetraacetic acid; FBS: Fetal bovine serum; GFP: Green fluorescence protein; L2R: Plasmid-free *C*. *trachomatis* L2 strain 2566R; PBS: Phosphate-buffered saline; RB: Reticulate body.

## Competing interests

The authors declare that they have no competing interests.

## Authors’ contributions

HF conceived the study. SX and XB constructed plasmids. SX and LB performed transformation and selection. SX performed iodine stain and acquired images. HF wrote the manuscript, which was reviewed and approved by all authors prior to submission. All authors read and approved the final manuscript.

## References

[B1] Schachter J Stephens RS Infection and disease epidemiology Chlamydia Intracellular Biology, Pathogenesis 1999 Washington DC: ASM Press 139 169

[B2] Campbell LA Kuo CC Chlamydia pneumoniae–an infectious risk factor for atherosclerosis? Nat Rev Microbiol 2004 2 1 23 32 10.1038/nrmicro796 15035006

[B3] Balin BJ Gerard HC Arking EJ Appelt DM Branigan PJ Abrams JT Whittum-Hudson JA Hudson AP Identification and localization of Chlamydia pneumoniae in the Alzheimer’s brain Med Microbiol Immunol (Berl) 1998 187 1 23 42 10.1007/s004300050071 9749980

[B4] Campbell LA Kuo CC Grayston JT Chlamydia pneumoniae and cardiovascular disease Emerg Infect Dis 1998 4 4 571 579 10.3201/eid0404.980407 9866733 PMC2640250

[B5] Hu H Pierce GN Zhong G The atherogenic effects of chlamydia are dependent on serum cholesterol and specific to Chlamydia pneumoniae J Clin Invest 1999 103 5 747 753 10.1172/JCI4582 10074493 PMC408120

[B6] Igietseme JU Eko FO Black CM Chlamydia vaccines: recent developments and the role of adjuvants in future formulations Expert Rev Vaccines 2011 10 11 1585 1596 10.1586/erv.11.139 22043957

[B7] Rockey DD Wang J Lei L Zhong G Chlamydia vaccine candidates and tools for chlamydial antigen discovery Expert Rev Vaccines 2009 8 10 1365 1377 10.1586/erv.09.98 19803759

[B8] Wang Y Kahane S Cutcliffe LT Skilton RJ Lambden PR Clarke IN Development of a transformation system for Chlamydia trachomatis: restoration of glycogen biosynthesis by acquisition of a plasmid shuttle vector PLoS Pathog 2011 7 9 e1002258 10.1371/journal.ppat.1002258 21966270 PMC3178582

[B9] Song L Carlson JH Whitmire WM Kari L Virtaneva K Sturdevant DE Watkins H Zhou B Sturdevant GL Porcella SF Chlamydia trachomatis plasmid-encoded Pgp4 is a transcriptional regulator of virulence-associated genes Infect Immun 2013 81 3 636 644 10.1128/IAI.01305-12 23319558 PMC3584862

[B10] Gong S Yang Z Lei L Shen L Zhong G Characterization of Chlamydia trachomatis plasmid-encoded open reading frames J Bacteriol 2013 195 17 3819 3826 10.1128/JB.00511-13 23794619 PMC3754608

[B11] Gerard HC Mishra MK Mao G Wang S Hali M Whittum-Hudson JA Kannan RM Hudson AP Dendrimer-enabled DNA delivery and transformation of Chlamydia pneumoniae Nanomedicine 2013 S1549-9634 13 00176 00177 10.1016/j.nano.2013.04.004 23639679

[B12] Peterson EM Markoff BA Schachter J De la Maza LM The 7.5-kb plasmid present in Chlamydia trachomatis is not essential for the growth of this microorganism Plasmid 1990 23 2 144 148 10.1016/0147-619X(90)90033-9 2362949

[B13] Caldwell HD Kromhout J Schachter J Purification and partial characterization of the major outer membrane protein of Chlamydia trachomatis Infect Immun 1981 31 3 1161 1176 7228399 10.1128/iai.31.3.1161-1176.1981 PMC351439

[B14] Bao X Nickels BE Fan H Chlamydia trachomatis protein GrgA activates transcription by contacting the nonconserved region of σ66 Proc Natl Acad Sci 2012 109 42 16870 16875 10.1073/pnas.1207300109 23027952 PMC3479454

[B15] Pachikara N Zhang H Pan Z Jin S Fan H Productive Chlamydia trachomatis lymphogranuloma venereum 434 infection in cells with augmented or inactivated autophagic activities FEMS Microbiol Lett 2009 292 2 240 249 10.1111/j.1574-6968.2009.01494.x 19187200 PMC2671565

[B16] Yasir M Pachikara ND Bao X Pan Z Fan H Regulation of chlamydial infection by host autophagy and vacuolar ATPase-bearing organelles Infect Immun 2011 79 10 4019 4028 10.1128/IAI.05308-11 21807906 PMC3187247

[B17] Li C-H Cheng Y-W Liao P-L Yang Y-T Kang J-J Chloramphenicol causes mitochondrial stress, decreases ATP biosynthesis, induces matrix metalloproteinase-13 expression, and solid-tumor cell invasion Toxicol Sci 2010 116 1 140 150 10.1093/toxsci/kfq085 20338993 PMC2886854

[B18] Suchland RJ Geisler WM Stamm WE Methodologies and cell lines used for antimicrobial susceptibility testing of Chlamydia spp Antimicrob Agents Chemother 2003 47 2 636 642 10.1128/AAC.47.2.636-642.2003 12543671 PMC151736

[B19] Wang Y Kahane S Cutcliffe LT Skilton RJ Lambden PR Persson K Bjartling C Clarke IN Genetic transformation of a clinical (genital tract), plasmid-free isolate of Chlamydia trachomatis: engineering the plasmid as a cloning vector PLoS One 2013 8 3 e59195 10.1371/journal.pone.0059195 23527131 PMC3601068

[B20] Carlson JH Whitmire WM Crane DD Wicke L Virtaneva K Sturdevant DE Kupko JJ 3rd Porcella SF Martinez-Orengo N Heinzen RA The Chlamydia trachomatis plasmid is a transcriptional regulator of chromosomal genes and a virulence factor Infect Immun 2008 76 6 2273 2283 10.1128/IAI.00102-08 18347045 PMC2423098

[B21] Tam JE Davis CH Wyrick PB Expression of recombinant DNA introduced into Chlamydia trachomatis by electroporation Can J Microbiol 1994 40 7 583 591 10.1139/m94-093 8076253

